# Multi-Source Remote Sensing Fusion and Deep Learning for Intelligent Precision Agriculture

**DOI:** 10.3390/s26206434

**Published:** 2026-10-12

**Authors:** Zhen Ma, Xiyuan Chen, Cundeng Wang

**Affiliations:** 1 School of Agricultural Engineering, Jiangsu University, Zhenjiang 212013, China; 1000005769@ujs.edu.cn; 2 School of Instrument Science and Engineering, Southeast University, Nanjing 210096, China

**Keywords:** precision agriculture, multi-source remote sensing, data fusion, intelligent perception

## Abstract

**Highlights:**

**What are the main findings?**

- Review of the integration architecture of multi-source remote sensing and deep learning for precision agriculture.
- Analysis of the complementary roles and limitations of optical, TIR, SAR, and LiDAR sensors.
- Summary of deep learning evolution from CNNs to spatiotemporal networks and Transformers.

**What are the implications of the main findings?**

- Provision of technical references for crop monitoring, crop trait estimation, and variable-rate management in complex agricultural environments.
- Support for intelligent decision-making through multi-source data fusion.

**Abstract:**

The fusion of multi-source remote sensing data and deep learning technology has been increasingly applied in precision agriculture. This review focuses on the perception–decision–execution framework and summarizes the current application status, technical routes, and development trends of multi-source remote sensing data fusion and deep learning in precision agriculture. It examines the complementary advantages of multimodal data such as optical, thermal infrared (TIR), Light Detection and Ranging (LiDAR), and synthetic aperture radar (SAR) in characterizing crop physiological traits and structural characteristics. At the algorithmic level, this review analyzes the evolution of deep learning architectures from single convolutional neural networks (CNNs) to spatiotemporal fusion networks (STFNs), Transformer-based architectures, and emerging state-space models, with emphasis on their capabilities for heterogeneous feature extraction and cross-modal information integration. This review discusses the transformation mechanism from intelligent perception to executable decision-making and explores the application effectiveness of prescription maps and variable-rate task technology in improving resource utilization. This review proposes forward-looking views on core bottlenecks such as model generalization, physical interpretability, and edge computing. This review not only provides a theoretical analysis of precision agriculture but also provides academic recommendations for the development of intelligent agricultural equipment in the future.

## 1. Introduction

Precision agriculture aims to improve agricultural productivity and reduce negative environmental impacts through optimized resource allocation and refined management. However, in complex and dynamically changing agricultural environments, the deep promotion and application of precision agriculture still face many practical challenges. The traditional method of obtaining agricultural information relies heavily on manual sampling and field investigations. This model is time-consuming, labor-intensive, and inefficient; furthermore, it cannot fully capture the high spatial heterogeneity exhibited by large areas of farmland in terms of soil physical and chemical properties, crop growth, and water distribution [1]. Faced with pest and disease outbreaks, spatially heterogeneous weed distribution, and complex non-point-source pollution, traditional monitoring methods often fail to meet the requirements of modern agriculture due to the lack of sufficient spatiotemporal resolution and rapid response capabilities [2,3]. It is urgent to introduce more efficient and intelligent Earth observation technologies to address the limitations of conventional agricultural monitoring.

**Figure 1:**
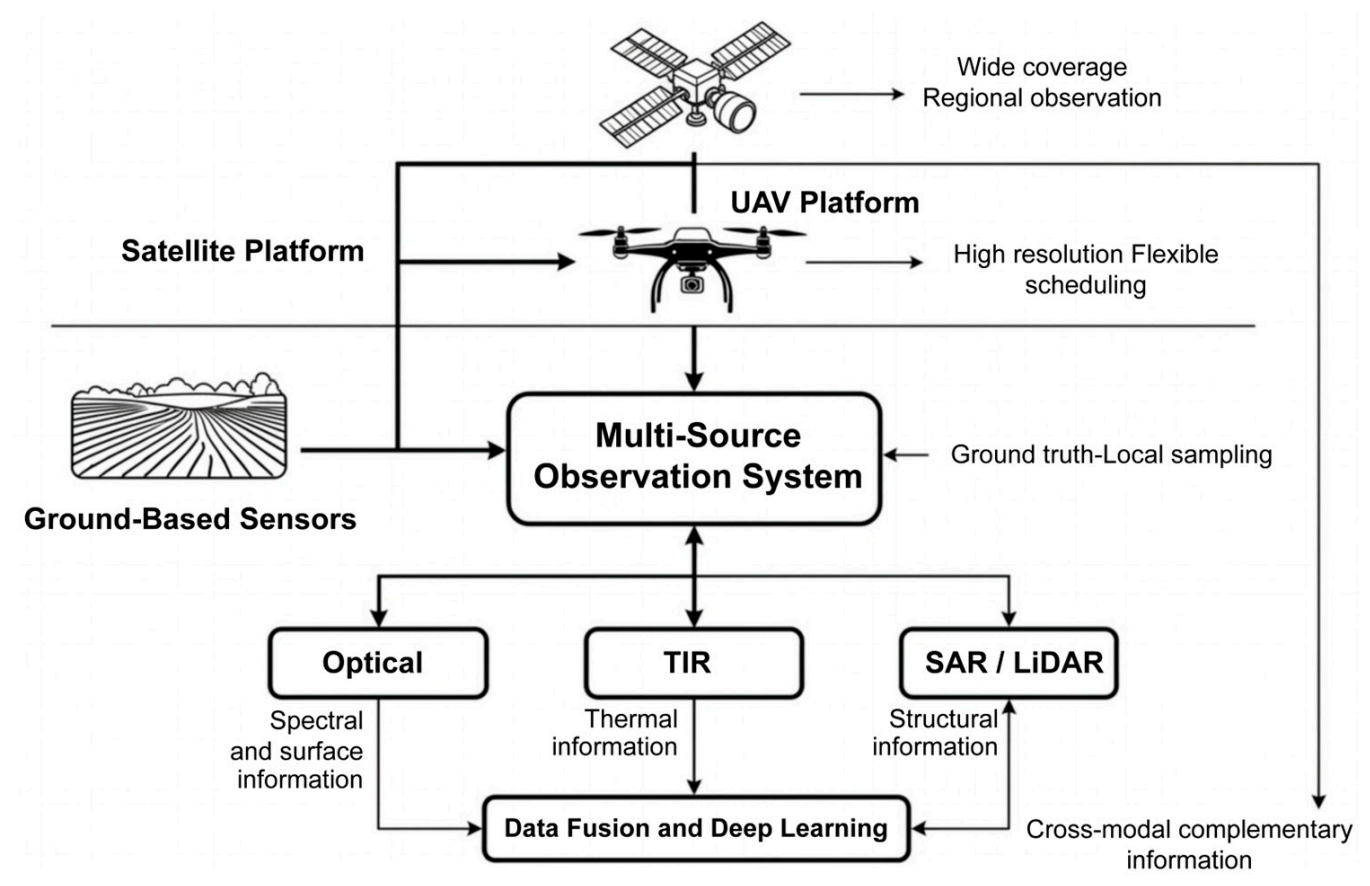
Multi-source observation and fusion framework for precision agriculture.

In recent years, with the rapid advancement of sensor hardware technology and aerospace platforms, the flexibility and high precision of Unmanned Aerial Vehicle (UAV) systems and the wide coverage capability of satellite platforms have achieved deep collaboration, promoting the development of multi-source remote sensing fusion technologies for intelligent agricultural monitoring. As shown in Figure 1, relying on the complementary advantages of sensors with different physical mechanisms such as optics, multispectral, hyperspectral, thermal infrared (TIR), and synthetic aperture radar (SAR), building a multi-layer perception system that integrates space–air–ground data has become an inevitable trend in modern agricultural dynamic monitoring [4]. At the same time, the rapid development of artificial intelligence, especially deep learning and advanced learning methods, has provided a powerful computing framework for extracting information from massive heterogeneous agricultural remote sensing data. Deep neural networks can not only alleviate some limitations associated with spectral confusion and environmental interference in single-source data under complex agricultural scenes but also extract spatial, temporal, and cross-modal representations, supporting high-precision analysis from crop phenotype analysis and growth monitoring to physical and chemical parameter inversion [5,6]. The integration of deep learning architectures with different feature fusion mechanisms has therefore provided new opportunities for improving the utilization of heterogeneous observations in agricultural remote sensing [7,8,9].

In recent years, numerous review studies have been published in the field of agricultural remote sensing, but these reviews differ substantially in terms of research scope and thematic emphasis. Some reviews (e.g., [10]) mainly focus on specific sensing platforms such as UAVs and micro/nano satellites and summarize developments in payload technologies and radiometric correction methods. These studies primarily emphasize sensor engineering and data acquisition technologies. Other reviews (e.g., [11]) focus on traditional machine learning algorithms and summarize their performance and limitations in crop classification tasks and local field physical parameter inversion. In addition, some recent reviews (e.g., [12]) have investigated the potential application of deep learning models in large-scale remote sensing data analysis, but the related research mainly remains focused on remote sensing-based perception and analysis, with relatively limited consideration of how heterogeneous remote sensing information can be further connected to agricultural decision-making and operational execution. This limitation highlights the need for a review framework that considers not only the accuracy of remote sensing interpretation but also the data coordination, fusion mechanisms, generalization conditions, and downstream usability of the resulting information.

Compared with previous reviews, this review establishes a comprehensive research framework covering three main stages: perception, decision-making, and execution. This review analyzes the physical complementary characteristics and applicable boundaries of multimodal remote sensing data used for agricultural monitoring and parameter estimation. An overview is provided of the iterative development process of deep learning models from CNNs, spatiotemporal fusion networks, and Transformer-based models to emerging state-space and Mamba-based architectures. Meanwhile, this review focuses on the key transition from heterogeneous observations to reliable agricultural decision-making information, including cross-source data coordination, multi-level fusion, and the conversion of remote sensing-derived information into prescription maps and other operational decision outputs. This review further examines the conditions under which multimodal fusion provides complementary advantages, as well as situations where noise, missing observations, domain shifts, or physical inconsistencies may reduce its effectiveness.

This review explores four closely related research questions: (1) How does the physical, spatial, spectral, and temporal heterogeneity between different remote sensing platforms and sensors affect data coordination and fusion? (2) Under what conditions can different fusion levels and deep learning architectures provide true complementary advantages, rather than just increasing model complexity? (3) How do noise, missing observations, scale differences, and domain shifts affect the reliability and generalization of multi-source fusion models in agricultural environments? (4) How can the fused remote sensing information be further transformed into actionable agricultural decisions and operational outputs? Based on these questions, this review aims to determine the current technological boundaries and future research directions of multi-source remote sensing fusion and deep learning in precision agriculture. This review emphasizes the conditions, mechanisms, and limitations that determine whether fused information can be reliably transformed into actionable agricultural decisions. This review considers studies published from 2018 to April 2026, with particular emphasis on recent developments during 2020–2026.

## 2. Multimodal Perception and Data Harmonization

**Figure 2:**
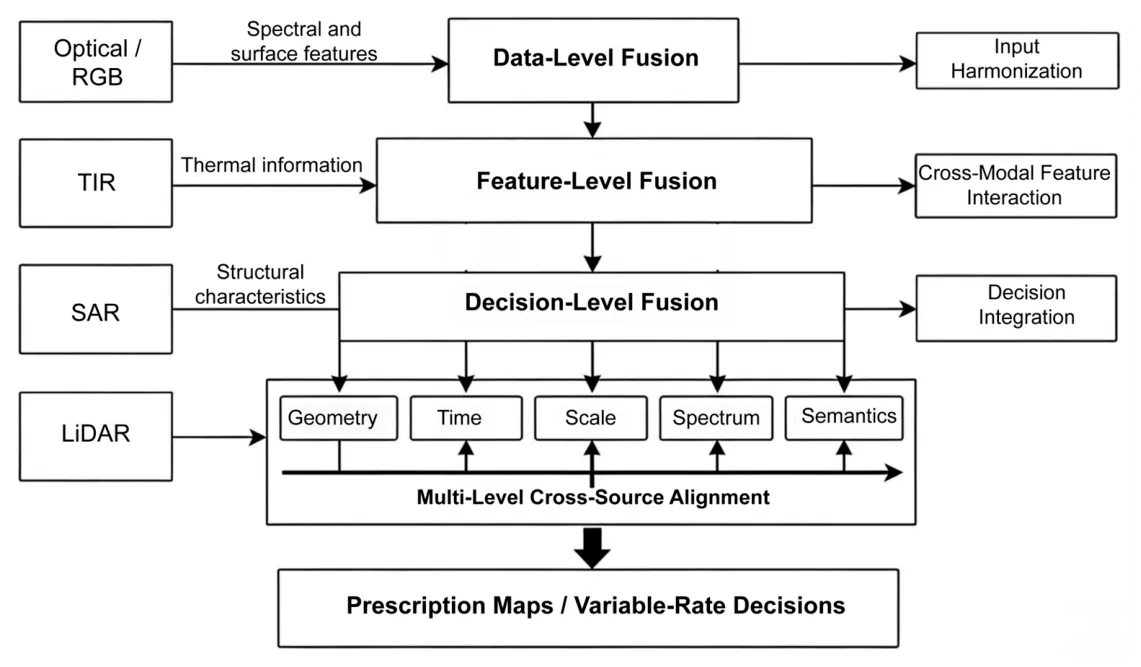
Multimodal data harmonization and fusion framework.

Precision agriculture relies on high-precision physical characterization of farmland systems, and a single data source is limited by spatiotemporal resolution and observation mechanisms, making it difficult to comprehensively characterize crop physiological states. As shown in Figure 2, this section explores the cross-scale integration mechanisms between UAVs and satellite platforms from two dimensions: platform-scale collaboration and multimodal sensor complementarity. It also analyzes the fusion potential and application limitations of multimodal heterogeneous data such as optical, TIR, SAR, and LiDAR.

### 2.1. Cross-Scale Integration of UAV and Satellite Platforms

Satellite remote sensing has a wide coverage area, but its spatial resolution and revisit period are limited by weather conditions such as cloud cover. UAVs have high spatial resolution and flexible scheduling capabilities, which can effectively compensate for this deficiency. In terms of cross-scale information transmission and data augmentation, UAV-derived data are often used as a reference benchmark to train and calibrate satellite estimation models. By combining algorithms such as Random Forest (RF) algorithms, this upscaling framework can explain 73% of the spatial variation in vegetation cover in heterogeneous agricultural landscapes, with a Root Mean Square Error (RMSE) of 12.13% [13]. Faced with the problem of sample scarcity, the collaboration of multi-source satellite data and the introduction of minority oversampling techniques have become effective approaches, which can increase the model’s Coefficient of Determination (R^2^) by 0.09 to 0.28 and reduce the RMSE by 0.30 to 38.57 g/m^2^ [14].

In response to the demand for multi-temporal dynamic monitoring in complex environments, the fusion of multi-period UAV multispectral, TIR, and LiDAR features, combined with segmentation models and classifiers, can achieve an accuracy of over 90% in early farmland planting structure extraction [15]. Researchers have used environmental datasets and multi-source remote sensing to construct knowledge-guided machine learning methods, which can generate annual spatial distribution maps of winter wheat at a resolution of 30 m, with an overall accuracy of 0.929 and an F_1_-score of 0.906, proving that the fusion of active and passive satellite platforms can enhance robustness in complex environments [16].

When comparing observation accuracies and field-scale deployments, UAV-derived surface temperature data exhibits high precision, serving as a valuable supplement for site-specific management [17]. When combining field sampling and remote sensing data to evaluate soil salinization, there is a correlation between vegetation coverage and salinity index, highlighting the cost advantage of remote sensing technology [18]. The low-cost quadcopter UAV platform combined with the Active Disturbance Rejection Control (ADRC) algorithm reduces trajectory control error to below 1.5 m, with a Normalized Difference Vegetation Index (NDVI) error of only 1.99%, meeting the requirements of low-altitude remote sensing [19].

Cross-scale platform fusion has significant utility in connecting micro-physiological features with macro-agricultural models, especially suitable for agricultural environments with high spatial heterogeneity. This type of fusion typically uses UAV data as a bridge between ground truth and satellite images. The applicability of this type of method is constrained by strict boundary conditions. The differences in physical characteristics of sensors on different platforms, spatial registration errors, inconsistent radiometric corrections, and time lag effects caused by data acquisition time differences can easily offset the accuracy benefits brought by algorithms. Existing benchmark tests are mostly conducted under controlled lighting and ideal weather conditions. When the model faces extreme weather or frequent cloud cover areas, maintaining cross-scale transformation robustness and controlling data processing costs for large-scale promotion remain the main challenges faced in practical implementation.

### 2.2. Complementary Physical Mechanisms of Multimodal Sensors

Multimodal fusion of sensors based on different physical mechanisms can overcome the limitations of single spectral observation and achieve comprehensive analysis of the internal physiological state and external three-dimensional structure of crops. Optical sensors are responsible for obtaining surface texture and pigment absorption characteristics. TIR sensors are highly sensitive to canopy transpiration and water stress. SAR and LiDAR can penetrate the canopy to obtain geometric structure and biomass volume information.

To analyze water stress and temperature characteristics, synchronously obtaining multispectral and TIR images has emerged as an effective approach to monitoring drought. The Temperature Vegetation Drought Index, derived from NDVI and the RGB-based atmospheric impedance index, can accurately capture the high-resolution spatial heterogeneity of crop water stress [20]. Faced with agricultural scenes with drastic changes in lighting conditions, a light–shadow separation model was constructed by combining 3 cm resolution RGB and 16 cm resolution multispectral images, with a Kappa coefficient of up to 0.93. This mechanism quantifies the physical process of a 2.4 °C increase in brightness temperature and a 26% decrease in NDVI during the transition from shadow to sunny conditions [21]. For low light conditions, the fusion of RGB images and TIR radiation information can enhance the boundary clarity and noise resistance of UAV image semantic segmentation [22].

A three-dimensional fusion framework covering phenotype, management, and environmental factors can be constructed by coupling multispectral indices, image planting density features, and LiDAR terrain slope information for the three-dimensional joint perception of crop structure and biomass. This framework achieved R^2^ values of 0.93, 0.90, and 0.92 in estimating aboveground biomass of maize at different growth stages [23]. In areas where optical remote sensing is susceptible to cloud and rain limitations, the fusion of optical and radar sensors provides complementary capabilities. By using a deep sparse autoencoder to fuse optical and airborne radar data in the feature layer, the sensitivity of radar to dielectric constant and canopy structure is utilized, resulting in a maximum increase of 20% in F-scores for oat and wheat categories [24]. By comprehensively extracting optical multi-phase features and radar backscatter features, the overall accuracy of fine-grained crop classification with complex planting structures reached 96.40%, with a Kappa coefficient of 0.93 [25].

The multi-source data fusion strategy has also been extended to the field of agricultural environment and health monitoring. Integrated spectroscopy and hyperspectral imaging can enhance the sensitivity of detecting food pollutants such as pesticide residues [26]. The combination of hyperspectral and UAV remote sensing has become the digital core of smart agriculture, and multimodal fusion and portable sensing systems are the future trends [27]. The strawberry health monitoring model based on the integration of TIR and the Internet of Things (IoT) environment has an accuracy rate of 86.9%, which is 30% higher than the single model, and is suitable for disease early warning [28]. The model prediction accuracy R^2^ of integrating multiple sensors to evaluate the pigsty environment achieved an R^2^ of 0.9086, effectively reducing the environmental stress risk of pigs through adaptive ventilation control [29].

### 2.3. Data Harmonization and Cross-Source Alignment

There are significant differences in spatial resolution, observation time, spectral response, geometric positioning, and data expression forms among multi-source remote sensing data, so different data sources cannot be effectively fused directly. Data harmonization and cross-source alignment are key steps in multi-source remote sensing fusion, from data stacking to effective information complementarity. Geometric and temporal consistency is the foundation of cross-source fusion. The differences in imaging time and observation conditions among different platforms can result in inconsistencies in the location, surface condition, and texture features of land objects. Regarding the cross-source matching problem between optical satellite images and ICESat-2 elevation data, previous studies have used terrain profile elevation sequence similarity for matching and reduced the horizontal and vertical positioning errors of large-scale images through weighted joint adjustment, indicating that geometric matching accuracy directly affects the reliable correlation of cross-source information [30]. Similarly, the MS2-UAV benchmark performs geometric normalization on RGB, thermal infrared, and multispectral data by unifying map projection, orientation, and scale and compares the matching performance of different modalities under multi-temporal conditions, further demonstrating that a unified spatial reference framework is an important prerequisite for evaluating and implementing cross-sensor fusion [31].

The differences in spatial scale and spectral characteristics need to be coordinated before fusion. High-resolution images typically have richer spatial structures, while medium- to low-resolution data have stronger spectral or regional coverage capabilities. SSF-TransUNet utilizes meter-level GF-2 imagery and Sentinel-2 multispectral data to construct a cross-source spatial–spectral joint model, reflecting the need for explicit modeling of the synergistic use between different spatial resolutions and spectral features [32]. In soil attribute prediction, UAVs have different spatial support and feature expressions compared to Sentinel-2, Sentinel-1, and terrain data. Relevant studies have achieved cross-scale feature extraction and alignment through multi-level wavelet decomposition and channel reweighting. After fusing UAV and satellite data, the average R^2^ was improved by 0.05 and the RMSE was reduced by 0.98 g kg^−1^ [33]. In addition, there may be differences in band range and spectral response among different data sources. Cross-source spectroscopic studies unify the effective bands of public and laboratory data to 1180–1460 nm through band alignment and selection and combine Savitzky–Golay with baseline correction to reduce the impact of cross-source differences on model migration [34].

High-level features and semantic consistency are also important aspects of cross-source coordination. Although RGB, multispectral, and vegetation index data are related in origin, their information expression has significant differences. Therefore, related studies use cross-source semantic alignment modules to achieve feature coupling between different representation spaces [35]. The fusion research of UAV images, trap data, and environmental sensor data also introduces a cross-modal feature alignment mechanism to construct a unified multi-source feature representation space [36]. For modalities with greater differences in physical mechanisms, such as optics and SAR, relying solely on simple concatenation may not fully utilize their complementary information. Therefore, it is necessary to achieve heterogeneous information coordination through feature space construction and adaptive fusion [37]. In addition, different land use products may also have semantic conflicts caused by inconsistent classification systems, boundary rules, and mapping scales. Relevant research further adopts conflict perception and confidence adaptive strategies to improve the consistency of multi-source products [38,39]. Therefore, the coordination of multi-source remote sensing data should be further extended from simple geometric registration and resampling to multi-level alignment across temporal, spatial, spectral, feature, and semantic dimensions, providing a consistent data foundation for subsequent deep fusion and cross-regional applications.

Existing research has shown that the effectiveness of multimodal fusion depends strongly on the consistency between sensing characteristics and application conditions. These methods are highly dependent on specific crop growth stages and environmental background conditions. TIR data is highly sensitive to environmental wind speed and soil exposure, while radar data is susceptible to coherent speckle noise during processing in rainy or high-humidity environments. Most reported studies demonstrate high validation accuracy under relatively controlled conditions, but in real, complex agricultural environments lacking independent large-sample validation sets, the generalization ability of some feature-level fusion models declines. Clarifying under what boundary conditions different modal data may conflict or even fail is a key issue that urgently needs to be addressed in the future. To compare the applicability and performance differences of different platforms and multimodal sensor combinations in agricultural remote sensing, Table 1 summarizes the representative applications and advantages of typical fusion schemes in different task scenarios.

Table 1 shows that the effectiveness of multi-platform and multimodal fusion largely depends on the relationship between sensing characteristics and application targets, rather than the fusion strategy itself. The cross-platform fusion between unmanned aerial vehicles and satellite observations mainly aims to balance the spatiotemporal scale: unmanned aerial vehicle platforms provide fine-grained structural information, while satellites provide high-frequency regional-scale observations. In contrast, cross-modal fusion enhances the robustness of the system by utilizing complementary physical mechanisms such as penetration ability, moisture sensitivity, and canopy temperature response. However, the performance improvement reported in existing studies cannot be directly regarded as a universal advantage of fusion methods, as evaluation metrics are heavily influenced by crop types, environmental conditions, spatial resolution mismatches, and validation strategies. For example, when prediction targets are mainly controlled by regional-scale phenological information, the performance of high-resolution UAV data may not necessarily be better than satellite observations. Subsequent fusion research should go beyond simple data combinations and shift towards research on adaptive feature alignment, uncertainty quantification, and cross-regional generalization ability.

## 3. Deep Learning-Driven Fusion Mechanisms and Algorithmic Evolution

### 3.1. Fusion Hierarchical Architecture

**Figure 3:**
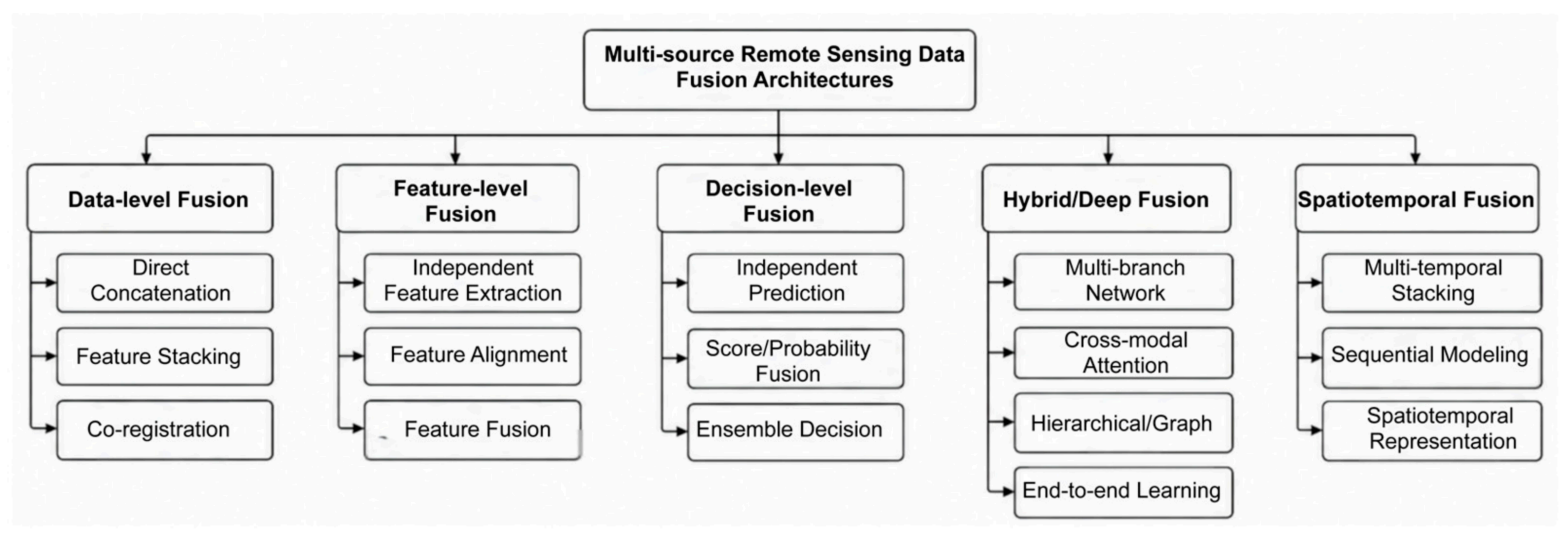
Classification of multi-source remote sensing data fusion algorithm architectures.

Multi-source remote sensing data fusion can be divided into data-level, feature-level, and decision-level fusion based on the depth of information interaction between different modalities. Figure 3 presents a classification framework for multi-source remote sensing data fusion algorithm architectures. Data-level fusion directly integrates multi-source data at the input stage, which can preserve the original spatial and spectral information relatively completely, but requires high spatial registration and scale consistency of multi-source data. Previous studies have shown that in low-overlap agricultural image processing, data-level processing combined with global texture information can achieve 3.4 fps while maintaining a 1.1-pixel reprojection error [50]. Feature-level fusion is a common strategy in current multi-source remote sensing research, which can enhance information complementarity between heterogeneous data through feature mapping and attention mechanisms. For example, semantic segmentation of UAV RGB images based on feature-level fusion can achieve over 90% mIoU, and deep fusion of different flight altitudes can achieve a Kappa coefficient of 0.92. To address the issue of missing multi-view data, teacher–student mutual learning and attention mechanisms can further improve the efficiency of cross-view feature fusion [51,52,53].

Decision-level fusion and its hybrid strategies mainly integrate multiple models or data sources at the level of prediction results or high-level semantic information. Related studies have shown that the hybrid fusion of handcrafted and deep features can alleviate the image degradation problem in rice blast resistance identification. The decision-level fusion of RGB and multispectral data can achieve an overall crop classification accuracy of over 90%. Object-level multi-source classification for mountainous environments also shows better performance than pixel-level methods [54,55,56]. In addition, the stacked ensemble learning of multispectral and TIR features, as well as meta-learning-based multimodal fusion, have been applied to soil salinity inversion and lettuce physiological status monitoring, respectively, further demonstrating that high-level decision fusion can improve model stability [57,58]. Overall, the choice of fusion level depends on data heterogeneity and task objectives: data-level fusion is beneficial for preserving underlying physical information but requires high registration and scale consistency. Feature-level fusion is more suitable for capturing cross-modal complementary relationships but usually has higher computational overhead. Decision-level fusion is more robust to single-modality data loss and noise but may lose the potential information gain brought by early cross-modal interactions.

### 3.2. Progress of Fusion Models Based on Deep Learning

#### 3.2.1. Evolution of CNN Architecture

CNNs are widely used to mine local spatial patterns and multi-scale texture features of remote sensing images. In crop classification and agronomic parameter inversion, researchers combined a hybrid framework of CNNs, time series models, and decision trees to classify nitrogen stress in winter wheat using multimodal UAV images, with an accuracy rate of 92.08% [59]. Combining autoencoders to extract deep features from optical and SAR images effectively reduces the extraction error of crop boundaries such as rice and wheat [60,61]. Multi-scale and attention mechanisms are introduced into the network architecture to address the issues of fragmented farmland plots and fuzzy boundaries. A semi-supervised dual-task architecture combining depthwise separable convolution and multi-scale attention is used to maintain high accuracy in wheat segmentation and counting under pseudo-label training [62]. The dual-branch model developed for temporal mapping of scattered farmland [63] and the fully convolutional network combined with morphological post-processing [64] effectively merge fragmented patches and improve the accuracy of farmland extraction. Improved object detection frameworks [65,66,67] that enhance local feature extraction capabilities in pest and small target recognition have improved localization accuracy under dense occlusion or background interference. The core advantage of CNN architecture in precision agriculture lies in its powerful local feature extraction and translation invariance, which is very suitable for processing farmland images with repeated textures. However, limited by a fixed receptive field, CNNs perform poorly in establishing global context dependencies for macroscopic land parcels. In addition, most CNN models are prone to overfitting when dealing with severe spectral overlap (such as extremely similar spectra between weeds and crops) or complex canopy occlusion in agricultural scenarios and rely heavily on large-scale, fine, labeled data.

#### 3.2.2. Spatiotemporal Fusion Network

The STFNs are mainly used to solve the trade-off problem between spatial resolution and temporal revisit frequency. In the application of a collaborative multi-model fusion strategy for cotton yield estimation in arid oasis areas, researchers reduced the computational cost of three-day data by 66.5% while maintaining low error [68]. The feasibility of deep learning networks in downscaling the spatial distribution of surface temperature products has also been thoroughly validated [69,70].

In terms of tracking crop phenological characteristics, the integration of UAVs and high-resolution satellite images has achieved high-precision inversion of water, salt, and carbon synergistic indicators during the growth period [71]. The spatiotemporal non-local filtering fusion model has shown good adaptability in simulating the growth curve of summer maize [72]. The constructed UAV satellite spatiotemporal fusion framework enhances the temporal resolution to the daily level [73]. By combining cross-modal transfer and phenological rhythms, the recognition accuracy of satellite time series in crop planting intensity and classification has been improved [74,75]. Optimizing the spectral variables of deep features has also effectively improved the non-destructive testing accuracy of hyperspectral rice variety recognition [76].

The spatiotemporal fusion network fills the data gap caused by cloud and rain cover in satellite images during key phenological periods of crops. The basic assumption is that the surface coverage exhibits gradual and predictable changes within the observation time window. This assumption is valid in the normal crop growth cycle, but when farmland encounters sudden disturbances (such as rainstorms and waterlogging, short-term mechanical harvesting, and pest outbreaks), existing models often cannot capture abrupt spectral changes and easily generate prediction images with smooth artifacts.

#### 3.2.3. Transformers and the Self-Attention Mechanism

The self-attention mechanism architecture provides a new paradigm for addressing global feature dependencies and cross-modal semantic alignment. The dual-branch vision framework replaces the standard matrix with a multi-head self-attention mechanism enhanced by convolution, achieving deep feature interaction and improving performance in crop recognition with high inter-class similarity [77]. The disaster detection framework that combines multispectral and RGB images with stride attention and cross-modal fusion performs better than traditional residual networks [78]. Microwave detection combined with a multi-task deep learning strategy has achieved quantitative analysis of wheat mold degree [79]. In recent years, Transformer-based architectures have attracted increasing attention in the field of agricultural remote sensing, mainly due to their ability to model long-range dependencies between heterogeneous spatial features. Unlike traditional convolutional neural networks (CNNs) that primarily rely on local receptive fields, Transformer models are able to capture global contextual relationships between multimodal observation data, which is highly advantageous for integrating optical, radar, hyperspectral, and elevation information. However, these advantages often come with higher computational requirements and a reliance on large-scale annotated datasets. Therefore, in actual deployment, a balance needs to be sought between representation capability, computational efficiency, and data availability.

#### 3.2.4. State Space Models and Mamba-Based Multimodal Fusion

In addition to the Transformer self-attention mechanism, the state space model (SSM) and Mamba architecture provide a new efficient path for long-range dependency modeling for multimodal remote sensing fusion. In response to the heterogeneity of hyperspectral and LiDAR data, GL-Mamba combines CNN, Transformer, cross-attention, and global–local Mamba modules while preserving local texture and global dependency information and exhibits good classification performance and inference efficiency on the Trento, Augsburg, and Houston2013 datasets [80]. Furthermore, HMGF-Net combines Mamba with graph fusion and multi-stage pseudo-label refinement for semi-supervised hyperspectral LiDAR classification under low-annotation conditions, showing good performance improvement even under 10% labeled-pixel conditions [81]. Regarding the complementary information between optical images and DSM, HACMNet utilizes adaptive dual-stream Mamba for cross-modal feature interaction to enhance the global semantic and spatial structural representation of complex terrain [82]. S2MSMamba further combines CNN local feature extraction, multi-scale hierarchical alignment, and Mamba long-range modeling and introduces adaptive decision-level fusion to improve hyperspectral LiDAR classification performance [83]. These studies indicate that the potential advantage of Mamba is not simply to replace Transformer-based models but to model long-range dependencies with lower computational complexity and complement CNNs, graph networks, and cross-modal attention.

#### 3.2.5. Input Noise and Data Quality in Deep Fusion

The effectiveness of deep fusion models is also strongly influenced by the quality of the input data. In agricultural remote sensing, heterogeneous observations may contain Gaussian noise, impulse noise, stripe noise, missing information, or other sensor-specific disturbances that can propagate through fusion networks and affect downstream interpretation. For hyperspectral images, He et al. developed a local low-rank matrix recovery framework with global spatial–spectral total variation to jointly separate low-rank clean information and sparse noise, demonstrating the importance of utilizing local low-rank structures and global spatial–spectral consistency in the restoration process [84]. Zhang et al. further combined nonlocal low-rank tensor decomposition with total variation regularization to solve the problem of mixed noise while maintaining nonlocal similarity and spectral correlation [85]. Peng et al. introduced a nonconvex low-rank and sparse separation framework with spatial–spectral total variation regularization, which was evaluated on simulated and real hyperspectral datasets [86]. Zhang et al. also proposed a sparsity-constrained low-rank approximation framework for handling heterogeneous Gaussian, impulse, and stripe noise, and experimental results showed improvements in quantitative and visual assessments [87]. These studies indicate that noise suppression is not simply a preprocessing step; maintaining spatial and spectral consistency and preserving informative structures are crucial for multimodal feature extraction and fusion. However, most existing studies only evaluate the denoising effect and lack a comparison of downstream fusion performance under different input conditions. Subsequently, a noise-aware evaluation framework needs to be constructed to comprehensively evaluate multiple performance indicators. Table 2 summarizes representative deep learning-based fusion architectures and their application performance in the field of agricultural remote sensing.

Table 2 shows that different deep learning architectures exhibit complementary advantages, rather than simply distinguishing between high and low performance. CNN-based models still maintain competitiveness in extracting local texture and spectral features, especially in scenarios with limited training samples. Transformer-based models have stronger global dependency modeling and cross-modal interaction capabilities, but their advantages often come with higher computational costs and data requirements. The spatiotemporal fusion network further enhances temporal consistency by utilizing phenological information from multi-temporal observations. However, most existing evaluations are based on benchmark datasets or limited experimental areas, and their robustness in large-scale, cross-year agricultural environments still lacks sufficient validation.

### 3.3. Lightweight and Real-Time Edge Computing

When converting deep perception information into instructions for agricultural machinery terminals to execute, the large number of parameters in the model often becomes a bottleneck for real-time computing. The current research not only focuses on algorithm compression but also emphasizes the deployment performance of models on resource-constrained platforms. In terms of algorithm lightweighting, researchers have developed an adaptive amplitude pruning algorithm for real-time weed recognition models using UAV remote sensing, which can reduce the number of parameters by 72.15% and increase the detection speed to 80.64 fps [104]. The lightweight cotton maturity recognition model has a size of only 0.35 MB and inference time reduced to 33 ms, maintaining a high accuracy of 0.96 in leaf shedding rate classification [105]. By compressing input dimensions and replacing convolutional layers with graph learning, feature redundancy is effectively eliminated, ensuring the inference speed of edge devices [106,107]. Improved pooling strategies and adaptive scaling parameters have improved end-to-end inference efficiency while maintaining high accuracy [108,109,110,111]. In response to the deployment limitations of picking robots and other equipment, the lightweight object detection model has achieved a detection speed of 65 fps [112].

At present, most literature focuses on reporting the theoretical computational load under ideal workstations, while ignoring the physical limitations in real agricultural scenarios. In actual deployment, edge computing nodes such as tractors or UAVs face strict power consumption constraints, poor heat dissipation, and bandwidth bottlenecks due to multi-source data synchronization. Although pruning and knowledge distillation techniques have reduced the model size, the robustness of the model to sudden changes in farmland lighting and motion blur will undergo nonlinear degradation under low-precision quantization. Designing a hardware-aware multimodal fusion architecture for specific agricultural machinery chips to achieve the optimal solution of computing power and accuracy is one of the biggest obstacles to breaking through the closed loop.

## 4. Multi-Source Perception Practice for Specific Agricultural Applications

The combination of multi-source remote sensing and deep learning provides technical support for solving specific agricultural problems. By synchronously perceiving multidimensional features, this technology model supports precise management throughout the entire phenological period of crops. This section explores the specific applications of multi-source perception in core fields such as crop physiological parameter inversion, stress recognition, phenotype evaluation, and yield prediction and objectively evaluates the performance gains and applicable boundaries of fusion technology in different practical scenarios.

### 4.1. Crop Growth and Physicochemical Parameter Inversion

Quantitative characterization of crop growth status is a prerequisite for achieving precise fertilization. Introducing structural features and texture information is the main solution to overcome limitations caused by spectral saturation. Machine learning techniques combined with feature inversion have been utilized through multi-organ feature selection models to integrate vegetation index and textural and morphological features to estimate aboveground biomass of rice. The validation R^2^ reached 0.89, which is better than traditional single spectral models [113]. Integrating RGB images and multispectral features of UAVs and combining texture features to invert nitrogen concentration in rice leaves, the regression model based on maximum correlation and minimum redundancy feature selection achieved an R^2^ of 0.68 at the jointing stage [114]. Researchers constructed a feature fusion network framework to integrate multi-source UAV features, resulting in a 36.67% improvement in the accuracy of estimating winter wheat leaf area index compared to a single RGB image model [115]. RF-based fusion of multiple spectral features can be utilized to control the prediction error of plant height from flowering to maturity within 1.01 cm [116]. The R^2^ value of the moisture estimation model constructed by researchers using hyperspectral imaging combined with adaptive sampling and feature selection is 0.713 [117]. The assimilation of physical models and remote sensing data provides a new approach to solving the problem of poor generalization ability in small sample data. Coupling crop growth models with hyperspectral images for predicting phenotype parameters showed high stability under small sample conditions, with an R^2^ of 0.86 [118].

Overall, the joint modeling of spectral information with three-dimensional structure, texture, and environmental variables can effectively alleviate the saturation problem of single spectral data in the mid-to-late-growth stage. However, currently, most models still rely on training samples collected in specific regions and years, and their performance improvement largely comes from optimization under the condition of consistent data distribution. When the model is migrated to different regions, varieties, or climatic conditions, it is often necessary to collect ground measurement data again for calibration. In addition, although the method based on crop growth models and remote sensing data fusion has stronger physical interpretation ability and spatiotemporal migration potential, the difficulty in obtaining complex parameters and high computational costs limit its large-scale practical deployment in agricultural production. Therefore, future research needs to shift from simply improving inversion accuracy to enhancing the model’s cross-regional generalization ability and practical deployment capability.

### 4.2. Accurate Diagnosis of Biotic and Abiotic Stress

Quickly identifying stress factors such as pests, diseases, and drought is an important step in reducing agricultural production losses. Multi-source perception technology provides a new solution for early diagnosis in complex stress environments by integrating information from different spatial scales and physical response mechanisms. The optimal spatial resolution for identifying wheat Fusarium head blight using multi-scale UAV remote sensing was determined to be 3.47 cm. The prediction accuracy of the regression model based on multi-source fusion reached 0.83 [119]. The cotton leaf mite detection system with integrated multispectral monitoring reported an accuracy of 85.47% using the RF algorithm [120]. The grape leaf disease diagnosis method that integrates deep features and classifiers achieves fast detection by concatenating dual network features, with a single image inference time of less than 1 s [121].

For abiotic stress monitoring, the combination of TIR imaging and multispectral analysis is the main method for evaluating water deficit. Research on drought monitoring based on UAV multispectral data shows that the constructed nonlinear regression model has an inversion R^2^ of 0.725 for leaf moisture content, which can be used to estimate the dynamics of deep soil moisture [122]. To improve the physical rationality of soil moisture estimation, the combination of a physics-driven neural network and a data augmentation estimation framework was used to integrate multi-source data, and the RMSE was controlled at 0.0349 [123].

Although multi-source fusion methods can achieve high detection accuracy under a single stress condition, pests, diseases, nutrient deficiencies, and environmental stresses in actual agricultural environments often have coexisting characteristics, and different stress factors may produce highly similar spectral and phenotypic responses. At present, most research is still based on relatively idealized experimental conditions, lacking long-term, multi-regional validation in complex field environments. Therefore, future research needs to focus on solving the problem of stress factor decoupling under multi-factor coupling conditions and improving the adaptability of models to unknown environments and new stress patterns.

### 4.3. Yield Prediction and Phenotype Analysis

Yield prediction is the terminal link of precision agriculture decision-making. Its accuracy depends on the deep integration of spatial, multi-source data and temporal information. When integrating agronomic traits and multi-temporal characteristics, the field-scale maize yield estimation model that integrates agronomic traits and spectral characteristics shows that combining multiple indicators yielded an R^2^ of 0.69 [124]. The sequence model for processing multi-temporal UAV images integrates color and spatial structure features to predict wheat biomass, with a maturity R^2^ of 0.527 [125]. Using high-resolution satellite time series to monitor rice yield under wheat residue cover, it was found that specific vegetation indices have the highest correlation with yield prediction [126].

In terms of introducing three-dimensional surface information and model calibration, evidence suggests that the R^2^ of the rice harvest index prediction model integrating digital surface elevation and spectral reflectance can reach 0.88 [127]. The volume yield regression model established by using LiDAR data combined with an improved 3D reconstruction algorithm to estimate rapeseed yield has a relative prediction error of about 12% at the field scale [128]. A process simulation model based on a crop growth model can reduce the relative error of biomass prediction during the grain-filling period to 3.44% by calibrating the jointing period data [129].

Overall, the accuracy of yield prediction usually improves as crops enter the grain-filling stage, as multi-temporal models can capture accumulated environmental and growth information throughout the entire growth cycle. Meanwhile, three-dimensional structural information provides a more direct physical basis for biomass estimation. However, there is still significant uncertainty in early yield prediction, mainly due to insufficient information on early crop phenotypes, environmental disturbances, and spatial errors in field yield measurement data. Therefore, future research needs to focus more on the reliability of models under independent regions and multi-year validation conditions, rather than simply pursuing high fitting accuracy on experimental datasets. To comprehensively compare the application of multi-source fusion technology in crop parameter inversion and yield prediction in recent years, Table 3 summarizes representative research results under different crops, target variables, data combinations, and evaluation indicators.

Table 3 shows that by integrating complementary spectral, structural, and environmental information, multi-source fusion technology can improve the performance of crop parameter inversion and yield estimation. However, the extent of this improvement largely depends on the specific application scenario. Models based on UAV data typically have higher accuracy due to their fine spatial resolution, while satellite-based methods demonstrate better scalability in regional applications. In addition, the differences in crop types, growth stages, feature engineering strategies, and validation schemes make it inappropriate to directly compare R^2^ or RMSE values in different studies. Therefore, future research should focus on developing models with uncertainty assessment capabilities and better generalizability, rather than solely pursuing maximum accuracy under specific experimental conditions.

## 5. Collaborative Decision-Making of Agricultural Machinery Terminals Driven by Data Fusion

**Figure 4:**
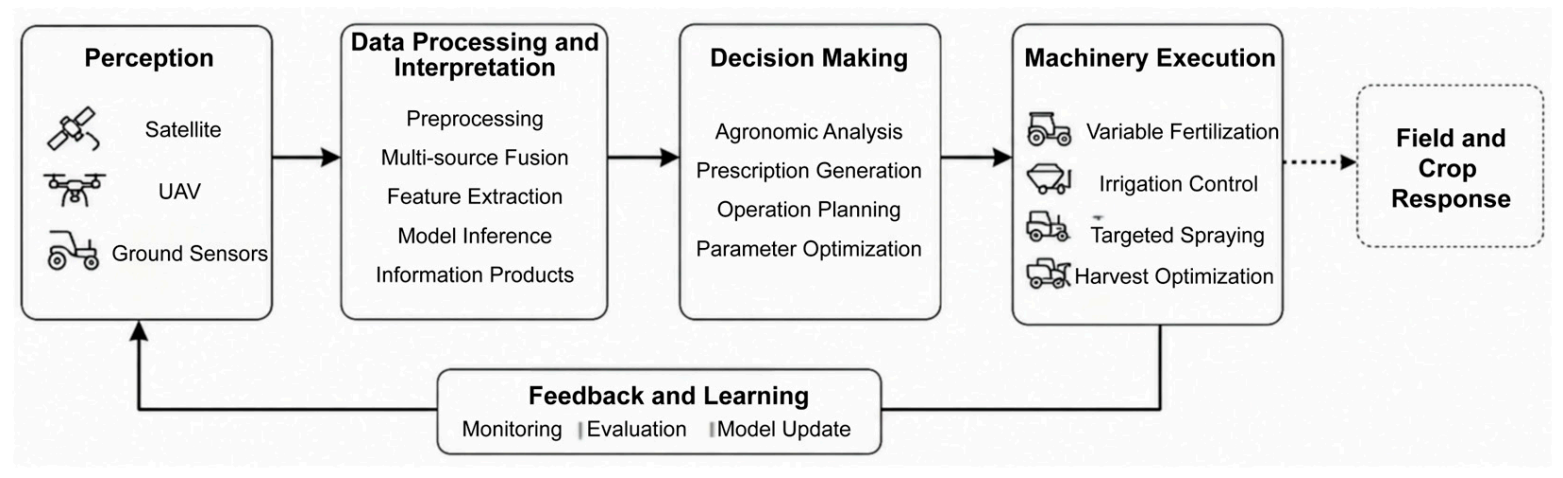
Closed-loop perception–decision–execution framework for precision agriculture.

The ultimate goal of precision agriculture is to transform remote sensing interpretation information into scientific decisions for agricultural machinery execution. The multi-source data fusion and deep learning inversion model mentioned earlier mainly outputs pixel-level agricultural parameters or classification probabilities. To achieve a closed-loop perception–analysis–execution system, it is necessary to reduce the dimensionality of these high-dimensional raster data and convert them into vector prescription maps or real-time control signals that can be directly read by agricultural machinery control systems. This section focuses on how multimodal data can guide variable fertilization, precision irrigation, weed management, and optimization of agricultural machinery parameters before harvesting. To illustrate the relationship among perception, interpretation, decision-making, and execution, Figure 4 presents a closed-loop framework for precision agriculture. The system integrates multimodal perception, data interpretation, intelligent decision-making, and agricultural machinery execution. The framework demonstrates how continuous raster data obtained from multi-source remote sensing can be reduced in dimensionality and transformed into discrete vector control signals recognizable by agricultural machinery terminals through core algorithms. A continuous iterative learning feedback stream is formed through the response of field crops.

### 5.1. Intelligent Generation of Prescription Maps

Intelligent prescription maps are an important digital interface that connects farmland state perception and agricultural machinery execution. Their core task is to convert continuous spatial distribution information into management zones and control areas suitable for variable-rate operation equipment. The current research mainly focuses on extracting land parcel boundaries, dividing target areas, and generating management units with multi-source data assistance. In terms of prescription map generation and task area delineation, low-cost UAVs and image classification algorithms have been used by researchers to generate prescription maps using neural networks, which can reduce the herbicide application area to 3.75% to 18.92% [150]. The high-precision orchard mapping pipeline, driven by a collaborative object detection and image segmentation large model, achieved an R^2^ of 0.97 in crown boundary extraction [151]. By combining object detection algorithms with clustering analysis, vegetation detection achieved an accuracy of 90% [152].

The adaptability of spatial resolution directly affects the decision-making accuracy when dealing with fragmented farmland. The field-scale resolution framework indicates that the image resolution of crops such as wheat needs to be better than 2.5 m to preserve texture features. Researchers combined high-resolution data and deep segmentation models and achieved an average IoU of 88.51% in highly heterogeneous land parcels [153]. In response to the frequent occlusion of linear boundaries in complex agricultural landscapes, the high connectivity extraction network has improved the boundary connectivity of land parcel recognition through a two-stage framework [154]. Research has shown that boundary semantic fusion networks can improve the IoU by over 6% compared to benchmark methods when generating prescription maps for farmland plots [155].

The fine-tuning application of basic large-scale models in prescription map generation has also made progress. After combining high-resolution images and introducing a prompt generation module to optimize the segmentation model, the overall accuracy index was improved to 0.958 [156]. Researchers compared different satellites and found that the mapping accuracy of specific indices in coverage mapping can reach 86.53% [157]. Using multispectral inversion to determine the appropriate harvest period, it was found that specific bands have the strongest correlation with thousand-grain weight. The constructed network model guides decisions to prevent harvest losses [158].

However, prescription map generation still faces the challenge of transitioning from “segmentation accuracy” to “operational feasibility”. At present, methods based on deep segmentation models such as the Segment Anything Model (SAM) and U-Net perform well in regular plots, but overlapping orchard canopies, irregular field ridges, and complex terrain can easily lead to topological errors in vector boundaries. Meanwhile, most studies still use image segmentation metrics such as mIoU and F_1_ to evaluate model performance, with less consideration given to file compatibility, region complexity, and control responsiveness of prescription maps imported into agricultural machinery. If the management area is divided too finely, it may exceed the response frequency of solenoid valves of variable-rate fertilizer applicators or spraying equipment. Therefore, future prescription map research needs to shift further from image segmentation accuracy to machine-executable optimization.

### 5.2. Variable Fertilization and Irrigation Control

The nutritional and water status of crops obtained from multi-source remote sensing inversion is an important basis for achieving variable fertilization and precise irrigation. However, the transition from remote sensing parameters to mechanical control variables still requires state estimation, decision optimization, and constraint transformation. In terms of nitrogen fertilizer variable management, the precise nitrogen management framework based on UAV multispectral diagnosis shows that variable fertilization within the plot can improve nitrogen fertilizer utilization efficiency by 19.02% to 20.11% compared to uniform fertilization throughout the field [159]. The optimization search algorithm combined with the adaptive segmentation framework estimates the tiller number by mixing spectral and texture shape features, and the model R^2^ can exceed 0.75, facilitating precision fertilization [160].

In terms of irrigation decision support, recent studies have demonstrated that based on the dual source energy balance model and UAV data estimation of evapotranspiration, the accuracy of net radiation estimation after integrating energy flux variation constraints reached 0.8501 [161]. After extracting sensitive bands using a water–nitrogen coupling decision model based on hyperspectral images, the R^2^ of the moisture estimation model reached 0.83, providing a path for dynamically adjusting water and nitrogen inputs [162]. The integrated method enabled root-zone moisture simulation at depths of 120 to 140 cm in multi-crop plots, with an R^2^ range of 0.76 to 0.91 [163]. The deep network model that integrates multi-source perception data to predict root zone moisture has an R2 of 0.868 on the validation set, effectively overcoming decision bias caused by spectral saturation [164].

From the perspective of device integration and scale transformation, real-time acquisition of crop water demand parameters through wireless sensor networks is fundamental to the implementation of water and fertilizer management decisions [165]. The comparison of instantaneous to daily-scale evapotranspiration upscaling methods provides theoretical support for multi-platform irrigation decision-making [166]. Optimizing fluid dynamics parameters can improve sprinkler uniformity and provide a reference for precision actuator design [167].

However, there are still significant scale differences between remote sensing inversion results and mechanical execution variables. Remote sensing mainly obtains canopy surface information, while fertilization and irrigation decisions are often influenced by root-zone water migration and nutrient dynamics. Although time-series models and environmental variables can partially compensate for this deficiency, they may still produce large errors under extreme drought, rainstorms, and other conditions. In addition, existing decision models usually assume that agricultural machinery has ideal execution capabilities and less frequently consider engineering factors such as nozzle pressure fluctuations, fluid response delays, and changes in operating speed. Therefore, future variable-rate operation systems need to further integrate agronomic models and mechanical dynamics models to achieve collaborative optimization of decision-making and execution.

### 5.3. Intelligent Weed Management and Targeted Spraying

Automatic weed recognition and targeted spraying require the model to not only accurately identify targets but also to generate spatial control information that matches nozzle control positions. Therefore, this task involves three levels simultaneously: target perception, spatial positioning, and real-time execution. In terms of weed mapping and detection network architecture, researchers have introduced an object detection model with wavelet convolution and an attention mechanism to improve detection speed while maintaining accuracy. The generated continuous density map is directly applied to variable spraying equipment, achieving a 3.4% increase in the growth rate [168]. The early monitoring system based on visual foundation models has an accuracy rate of over 99.1% in classifying various weeds, confirming the ability of self-attention architecture to distinguish crops from weeds [169]. Orchard targeted spraying technology places multi-source data fusion at the core of tree crown modeling and precision pesticide application [170]. In terms of obstacle avoidance for crop protection machines, the architecture combining airborne sensors and fluid dynamic data has successfully avoided vertical obstacles while achieving a spray coverage rate of over 98% [171].

Although visual models have high accuracy in static testing environments, in actual high-speed motion of agricultural machinery, image motion blur, lighting changes, and crop occlusion can lead to a decrease in model performance. Meanwhile, weeds and crops have highly similar phenotypic characteristics in the early stages of growth, and relying solely on two-dimensional visual information can easily lead to misjudgment. Therefore, the fusion of depth vision features and 3D structural information such as LiDAR, combined with real-time edge computing, is the key direction to improve the reliability of dynamic spraying systems.

### 5.4. Pre-Harvest Crop Condition Assessment and Harvest Machinery Optimization

Pre-harvest crop state prediction can provide decision support for optimizing harvesting machinery speed, header height, and operation path and is an important link connecting remote sensing monitoring and agricultural equipment control. In terms of state assessment and scheduling decision-making, researchers have achieved an accuracy of 0.83 in the residual coverage evaluation model by combining satellite features with true values [172]. The height modeling method based on UAV LiDAR extracts information with an R^2^ of 0.82, informing parameter adjustment of large-scale cotton picking machines [173]. The regression model that integrates RGB images and multispectral TIR features achieved a prediction accuracy of 0.918 for defoliation rate [174]. Adopting optimized ensemble learning algorithms to evaluate crop status and achieve an R^2^ of 0.887 provides guidance for optimizing agricultural machinery scheduling [175].

In terms of the integration of low-level control and perception in agricultural machinery, a harvester navigation control system based on visual Simultaneous Localization and Mapping (vSLAM) and inertial navigation fusion controls lateral deviation at the centimeter level [176]. Researchers used a multi-sensor fusion-based seeding depth control system to detect terrain features using LiDAR, reducing the average seeding error by 22.9% [177]. The sensor, enhanced via fuzzy inference fusion technology, reduces the particle measurement error to less than 9.2% [178]. The enhanced feature fusion network combined with depth cameras calculates the stem lodging angle, with an average error of only 5.364%, supplying actionable data for real-time adjustment of harvester parameters [179].

The deep integration of high-dimensional remote sensing information into the underlying execution loop of agricultural machinery equipment (e.g., harvester navigation and seeding depth control) marks the evolution of precision agriculture from a simple monitoring technology to a decision execution system with real-time feedback capability. At present, the main limitation is that most of the pre-harvest evaluation models rely on offline data processing (such as bringing the UAV orthophoto image back to the workstation for processing and generating routes), while real-time dynamic control systems based on VSLAM or edge computing cameras often have limited computing resources. How to achieve high-frequency spatiotemporal synchronization of multiple sensors (e.g., LiDAR, binocular vision, inertial navigation) under the limited computing power of agricultural machinery vehicles is the core technical challenge to enable the deployment of fully autonomous operation of agricultural machinery.

Overall, the integration of multi-source remote sensing information into the underlying control of agricultural machinery has promoted the development of precision agriculture from a “monitoring system” to a “decision-making and execution system”. However, at present, a large number of pre-harvest evaluation studies still rely on offline processing processes, such as generating work paths after UAV image retrieval, while methods based on edge computing and real-time vision systems are limited by on-board computing resources. Therefore, achieving high-frequency spatiotemporal synchronization and real-time decision-making of multiple sensors with limited computing power is a key challenge for the fully autonomous operation of future intelligent agricultural equipment. To compare representative studies from the perspective of actionable decision-making, Table 4 summarizes recent research based on application areas, integration strategies, operational performance, and corresponding agricultural machinery execution stages.

Overall, Table 4 indicates that multi-source data fusion technology is driving the development of precision agriculture from simple state monitoring to executable decision-making. However, the evaluation indicators in different studies are at different technical levels, including perception accuracy, decision effectiveness, and mechanical execution performance, which cannot be simply compared horizontally. Therefore, future research needs to establish a comprehensive evaluation system covering “perception reliability, decision effectiveness, and mechanical execution stability”.

At present, most studies still focus on improving the prediction accuracy of the algorithm, while the long-term stable operation of the complete closed-loop system in the real field environment is still limited by sensor synchronization error, communication delay, edge computing capability, and mechanical execution dynamic characteristics. The development focus of future agricultural intelligent equipment will shift from single-module performance optimization to collaborative optimization between multi-source perception, intelligent decision-making, and mechanical execution.

## 6. Challenges and Future Prospects

### 6.1. Multi-Source Observation Differences and Cross-Domain Generalization

Existing deep learning models typically rely on specific regions, years, crops, and sensor combinations, and their performance is easily affected by changes in illumination, crop varieties, soil background, growth stage, sensor configuration, and spatial resolution. For multi-source remote sensing, different modalities have different observation mechanisms and data distributions. For example, optical data is easily affected by cloud cover and illumination, TIR is affected by environmental temperature and soil exposure, and SAR has different scattering characteristics. When a modality has an obvious domain shift, its change may further propagate into the fusion feature space.

Therefore, domain-shift decomposition and adaptation methods for multi-source remote sensing should be developed to distinguish distribution changes caused by sensors, regions, seasons, growth stages, and crop types and dynamically adjust the contributions of different modalities. At the same time, cross-regional, cross-year, and cross-sensor validation should be strengthened to avoid relying solely on random data partitioning to achieve high accuracy.

### 6.2. Noise Propagation, Missing Modalities, and Error Coupling

Actual agricultural remote sensing data are easily affected by cloud cover, shadows, stripe noise, missing observations, and sensor failures. After entering the deep network, abnormal information may be further propagated through feature encoding, attention mechanisms, and cross-modal interactions, leading to erroneous information coupling in the fusion model. Especially when the quality of a certain modality decreases, the fixed fusion weight may limit the utilization of information from other reliable modalities.

In the future, a noise-aware fusion framework for downstream tasks should be established, combining noise detection, modality quality assessment, dynamic weight adjustment, and missing-modality reconstruction. When a sensor is unavailable or its reliability decreases, the model should automatically reduce its contribution and utilize other modalities to maintain basic perception capability, evolving from static fusion to quality-aware and fault-tolerant fusion.

### 6.3. Physical Inconsistency and Interpretability of Deep Fusion

Deep networks can learn complex cross-modal relationships, but high prediction accuracy does not necessarily mean that the model has learned stable agricultural physical principles. Optical data, TIR, SAR, and LiDAR have different physical observation mechanisms, and their relationships may vary with crop varieties, growth stage, canopy structure, and environmental conditions. If the model overly relies on statistical correlations in a specific dataset, its cross-regional generalizability may be limited.

Therefore, it is possible to further combine crop growth models, radiative transfer models, and soil process models; introduce constraints with clear physical meanings; and improve the physical consistency of the fusion representation. The physical priors applicable to different sensor combinations and agricultural tasks should be identified, and their impact on generalization capability and stability under abnormal conditions should be evaluated.

### 6.4. Computational Burden and Edge Deployment of Multimodal Fusion

Models such as Transformer, 3D convolution, cross-modal attention, and Mamba have improved multi-source fusion capabilities but also increased computational, storage, and data transmission burdens. Actual agricultural equipment is also limited by power consumption, heat dissipation, communication bandwidth, sensor synchronization, and spatial registration. Therefore, the deployment cost of multi-source fusion depends not only on the number of model parameters but also on the complete data processing chain.

We should strengthen the hardware–algorithm co-design; reduce computational burden through pruning, quantization, knowledge distillation, and lightweight networks; and optimize data transmission based on the acquisition frequency, resolution, and task importance of different modalities. Edge–cloud collaboration can also be used to balance model accuracy, real-time performance, power consumption, and communication costs.

### 6.5. Uncertainty Quantification and Failure-Aware Fusion

Current research mainly uses metrics such as accuracy, F1, R^2^, and RMSE to evaluate model performance, but these metrics may not indicate when the model may produce unreliable predictions. Disease identification, variable fertilization, and precision pesticide application in precision agriculture all have strong decision-making risks, so reporting only average accuracy is not sufficient to support practical applications.

Research should combine uncertainty quantification with multimodal fusion; estimate prediction reliability through Bayesian methods, evidential deep learning, and other techniques; and dynamically adjust fusion strategies based on sensor quality and model uncertainty. When there is obvious conflict between different modalities or the reliability of a certain modality decreases, the system should be able to perform modality down-weighting, modality switching, or re-perception to form a fusion system with quality awareness, fault detection, and dynamic reconfiguration capabilities.

### 6.6. Reproducibility, Economic Feasibility, and Technology Adoption

The practical adoption of multi-source fusion technology is still limited by sensor costs, data acquisition, maintenance, and equipment adaptation capabilities. Hyperspectral, LiDAR, and TIR can provide rich information, but not all agricultural tasks require high-cost sensors. Therefore, research should shift from maximizing the number of sensors to task-driven sensor configuration optimization and compare the comprehensive performance of different sensor combinations in terms of accuracy, computational cost, energy consumption, and agricultural benefits.

The current research still faces issues such as limited dataset size, inconsistent collection conditions, lack of unified standards for code and data preprocessing workflows, and difficulty in directly comparing results across studies. Therefore, it is necessary to establish a multi-regional, multi-year, and multimodal agricultural remote sensing benchmark dataset; unify data-quality descriptions, training and testing partitions, and evaluation indicators; and incorporate model accuracy, computational cost, sensor cost, and actual agricultural benefits into a unified evaluation framework to narrow the gap between academic model performance and agricultural production applications.

## 7. Conclusions

This review summarized recent advances in multi-source remote sensing data fusion and deep learning in precision agriculture and proposed an integrated framework encompassing multimodal perception to agricultural decision-making and operational execution. Research has shown that multi-source remote sensing data has evolved from relatively simple data-level integration to feature-level, decision-level, and deep cross-modal fusion. It increasingly emphasizes the complementary use of optical, TIR, SAR, LiDAR, and other heterogeneous observations. Spatiotemporal fusion networks, Transformer-based architectures, and emerging state space models provide additional capabilities for temporal modeling, global dependency modeling, and efficient long-range representation learning.

This study also suggests that higher model complexity or more input modalities do not necessarily lead to more reliable fusion performance. Differences in spatial and temporal resolution, sensor characteristics, noise, missing observations, and environmental conditions may introduce feature inconsistencies and error propagation during the fusion process. In addition, domain variations across regions, years, crop types, and sensing conditions remain the main obstacles to model generalization. Therefore, future research should go beyond accuracy-oriented model development and shift towards fusion strategies that can dynamically adapt to changes in data quality and sensing conditions, including noise-aware, uncertainty-aware, and physically consistent fusion.

Another challenge is to transform high-dimensional remote sensing information into actionable agricultural decisions and operational outputs. Prescription maps, crop physiological parameter estimation, disease monitoring, and other fusion-derived products can provide valuable decision-making information, but their practical value depends on their compatibility with downstream agricultural operations. Actual deployment requires careful consideration of model inference efficiency, sensor synchronization, communication latency, power consumption, and the physical constraints of agricultural machinery. Therefore, hardware-aware multimodal fusion, edge–cloud collaboration, and fault-aware decision-making mechanisms are important directions for narrowing the gap between remote-sensing-based perception and agricultural operations.

There are several limitations to this review. Due to significant differences in crop types, environmental conditions, spatiotemporal resolution, data sources, and validation protocols among the reviewed studies, direct quantitative comparisons cannot be made for all studies. Subsequent research should establish more standardized multi-regional and multi-year benchmarks to improve data quality and reporting on computational requirements. At the same time, the fusion methods need to be evaluated not only through prediction accuracy but also through robustness, generalization ability, computational efficiency, and practical agricultural benefits. These efforts will help promote the development of multi-source remote sensing fusion from high-performance experimental models to robust, scalable, and deployable precision agriculture systems.

## Figures and Tables

**Table 1:** Representative applications and performance of cross-platform and multimodal fusion in precision agriculture.

Fusion Type	Sensor/Data Combination	Research Objective	Quantitative Performance Indicators	Main Advantages	Ref.
Cross-platform radiometric consistency correction	Multimodal UAV Multispectral (MS)	Data consistency and radiometric calibration	RF-based sensor conversion achieved (R^2^) up to 0.91; improved vegetation index consistency after radiometric correction	Improved cross-domain vegetation index (VI) consistency	[40]
Cross-platform fusion	PlanetScope + UAV-MS	Winter wheat growth and fertilization monitoring	PlanetScope prediction: canopy cover (R^2^ = 0.68), yield (R^2^ = 0.54), protein content (R^2^ = 0.49); UAV models showed lower performance for some parameters	Balances large-scale survey potential with UAV high-res details	[41]
Cross-platform fusion	Sentinel-2 + UAV-MS	Vineyard yield and quality prediction	Prediction (R^2^ = 0.37–0.45) for grape yield and quality-related parameters	Cover-crop spectra aid prediction; new mechanism for satellite modeling	[42]
Cross-platform validation	UAV-MS + Multi-source Satellite	NDVI accuracy verification in heterogeneous landscapes	Sentinel-2 (R^2^) up to 0.91; RMSE down to 0.09	Effectively identifies and corrects systematic bias of mid-low-res satellites	[43]
Cross-modal comparison and integration	UAV microwave + TIR	Farmland soil moisture mapping	Soil moisture retrieval RMSE: 0.05–0.06 m^3^/m^3^ (L-band microwave); 0.05–0.09 m^3^/m^3^ (thermal-optical)	High sensitivity of microwave to moisture	[44]
Cross-modal fusion	UAV TIR + Ground Monitoring	Vineyard stress and heterogeneity analysis	Stress distribution mapping accuracy: 0.83	Enables high-precision spatial management	[45]
Cross-modal fusion	Sentinel-1 SAR + Sentinel-2 optical	Surface soil moisture estimation	RMSE: 0.023–0.024 cm^3^/cm^3^; (R^2^ = 0.886–0.891)	Improves estimation accuracy for low–mid vegetation	[46]
Cross-platform fusion	Multi-source satellite imagery (Sentinel-2/Landsat/GF)	Catchment-scale planting structure mapping	Overall accuracy: 80%	Overcomes revisit cycle limits	[47]
Cross-modal temporal fusion	Sentinel-1 SAR + Sentinel-2 optical time series	Multi-temporal crop classification	Accuracy improvement: 17.1% compared with single temporal observations	Tracks phenological growth stages; fills optical observation gaps caused by cloud cover	[48]
Cross-modal feature fusion	Sentinel-1 + Sentinel-2 deep feature fusion	Dual-channel CNN feature fusion	OA: 98.40%; Kappa: 0.98; Macro-F_1_: 0.98	Enhances differences between similar crops	[49]

**Table 2:** Representative deep learning-based feature fusion approaches for agricultural remote sensing applications.

Architecture Group	Model Architecture	Application Scenario	Performance Indicators	Validation Scope	Ref.
CNN-Based Models	CCSNet + multi-level feature fusion	GF-type maize canopy shadow/veg segmentation	mIoU = 86.42%; PA = 93.58%	Single dataset; single growth stage	[88]
	Minority-class augmentation + semantic segmentation	UAV fine-grained imbalanced crop classification	mIoU improvement: 1.54–7.08%	Two UAV datasets	[89]
	Adaptive SqueezeSlim U-Net	Resource-constrained real-time UAV weed detection	Energy consumption reduction: 65%; accuracy decrease < 2%	Two UAV datasets	[90]
	Machine learning + object-oriented ensemble	Spectral–texture (GLCM)-based crop identification	OA = 95%	Single UAV dataset	[91]
	GLCANet	Heterogeneous multi-source farmland boundary extraction	OA improvement = 3.2%; mIoU improvement = 2.6%	Multiple remote sensing datasets	[92]
	Attention-enhanced NIR-RGB-M2Net	Multi-source high-precision farmland segmentation	F_1_ = 94.54%; IoU = 89.65%	Two benchmark datasets	[93]
Transformer Models	Swin Transformer + local axial attention	Suburban/agricultural resource semantic segmentation	mIoU improvement = 0.8–3.6%	Multiple benchmark datasets	[94]
	Cross-domain FKIFM Transformer	UAV extreme-scale small object detection	mAP@0.5 improvement = 6.3%	Multiple UAV datasets	[95]
	Photo-aware cross-attention network	All-weather aerial tiny object recognition	mAP improvement > 3.6%	Multiple datasets	[96]
	Structured UniHSFormer-X	Heterogeneous HS fine-grained crop classification	OA = 99.80%; AA = 99.28%	Three UAV hyperspectral datasets	[97]
	Vision Transformer (ViT/Swin)	Complex background crop/weed classification	Accuracy = 91.65–92.97%	UAV images; single region	[98]
	Multi-scale CNN–Transformer	UAV global–local context crop segmentation	mIoU = 85.33%; PA = 92.46%	Benchmark remote sensing dataset	[99]
Spatiotemporal Fusion	SA-LSTM + feature transfer network	Spatiotemporal multi-source land use mapping	OA = 99.00%	Dataset-based evaluation	[100]
	3D U-Net + SE module	MS + SAR time-series seasonal mapping	OA = 94.5%	Regional seasonal mapping	[101]
	STFCropNet	Spatiotemporal denoising and crop classification	OA = 83.2–90.6%	Two regions; different seasons	[102]
	Multi-stream STGAN	Image fusion under drastic land surface changes	Improved SSIM and spectral fidelity	Multiple benchmark datasets	[103]

**Table 3:** Representative applications of multi-source remote sensing fusion for crop parameter inversion and yield estimation.

Crop Type	Multi-Source Data and Fusion Strategy	Validation Performance	Spatial and Temporal Validation	Ref.
Winter Wheat	UAV multispectral vegetation indices + texture features	AGB: R^2^ = 0.86, rRMSE = 27.47%; PNC: R^2^ = 0.82, rRMSE = 14.62%	UAV field experiment; single growing season	[130]
Rice	UAV RGB + multispectral imagery; color, texture, and vegetation index feature fusion with ensemble learning	R^2^ = 0.8564; RMSE = 169.32 g/m^2^	Multiple critical growth stages; UAV field observations	[131]
Lemongrass	MS vegetation indices	R^2^ = 0.79; RMSE = 0.05	UAV field monitoring; single crop system	[132]
Potato	FVC + CH	AGB: R^2^ = 0.834; yield: R^2^ = 0.792	Field experiments; single season	[133]
Peanut	UAV multispectral imagery + spectral indices + texture features	Plant height: R^2^ = 0.912, RMSE = 2.14 cm; SPAD: R^2^ = 0.530, RMSE = 3.87	Independent cross-year validation; four growth stages	[134]
Maize	Canopy temperature + water stress index	R^2^ = 0.84; RMSE = 242.5 g/m^2^	UAV monitoring under different irrigation treatments	[135]
Peanut	Spectra + texture + wavelet	R^2^ = 0.828	UAV multi-stage observation	[136]
Maize	Spectra + texture + CH	R^2^ = 0.88; RMSE = 0.35	Two experimental regions; multiple environments	[137]
Winter Wheat	Sentinel-1 (SAR) + Sentinel-2 (MS)	R^2^ = 0.731; RMSE = 2.2%	Regional agricultural area; multiple land covers	[138]
Winter Wheat	Spatiotemporal spectra	R^2^ = 0.66; RMSE = 0.61 t/ha	Multi-year satellite observation	[139]
Winter Wheat	MS + texture	R^2^ = 0.865; rRMSE = 16.3%	Multi-growth-stage UAV survey	[140]
Alfalfa	MS + environmental variables + CH	Yield: R^2^ = 0.921; RMSE = 300 kg/ha	Field experiment; single production cycle	[141]
Common Bean	VIs + texture	RMSE = 3.42 g/kg	Multi-year UAV experiment	[142]
Rice	VIs + deep texture	R^2^ = 0.96; RMSE = 813 kg/ha	UAV full growth cycle monitoring	[143]
Winter Wheat	RGB + MS	R^2^ = 0.725; RMSE = 0.814 t/ha	Multi-year UAV dataset	[144]
Spring Maize	UAV-MS vegetation indices	R^2^ = 0.99; RMSE = 0.30 cm	Multi-stage field experiment	[145]
Sugarcane	Multi-temporal sensitive spectra	R^2^ = 0.83; RMSE = 6.63 t/ha	Multi-stage UAV monitoring	[146]
Spring Wheat	VIs + LAI (structural feature)	R^2^ increased from 0.69 to 0.842	Different irrigation conditions	[147]
Winter Wheat	Multi-source remote sensing + ensemble learning	R^2^ = 0.76; rRMSE = 0.06	Multi-stage and multi-depth monitoring	[148]
Rapeseed	Multisensor vegetation indices	R^2^ = 0.814; RMSE = 171.9 kg/ha	UAV field experiment	[149]

**Table 4:** Multi-source data fusion applications for agricultural decision support and operation management.

Application Scenario	Fusion Algorithm and Model	Validation Scope	Perception Performance	Machinery Execution Links/Parameters	Ref.
Prescription mapping	Cascaded Sentinel-1/Sentinel-2 edge model	Northern Xinjiang; ~600 km^2^; multi-temporal satellite data	Improved farmland boundary extraction accuracy	Auto-navigation boundary vectors	[180]
	Attention-based U2-Net++	Multi-region satellite image patches	F_1_ = 97.13%	Guidance track control vectors	[181]
Precision fertilization	Multi-index RF/MLR/BP	Tomato field; multiple growth stages	LNC prediction accuracy > 0.8	Variable nitrogen fertilizer rate	[182]
	Stratified XGBoost/CNN	Maize under different water–nitrogen treatments	NNI R^2^ = 0.68; LNW R^2^ = 0.83	Top-dressing spatial grid maps	[183]
	Time-series dynamic + GIS	Three agroecological regions	RMSE reduction 43–70%	Variable-rate applicator file input	[184]
Intelligent plant protection	Self-Supervised Learning (SSL)	Soybean disease monitoring; single season	Accuracy/F_1_ = 0.92	Site-specific fungicide control	[185]
	Improved YOLOv4 + Tiny Mask	UAV maize dataset	mAP = 86.89%	Real-time nozzle switch trigger	[186]
	Air-ground spectroscopy fusion	Rice field; single season	Accuracy = 96.37%; Kappa = 0.95	Potential input for site-specific disease management	[187]
Pre-harvest assessment	Multi-temporal XGBoost	Cotton; multi-temporal UAV surveys	Yield prediction R^2^ = 0.802	Picker monitor yield calibration	[188]
	GPR with RGB + hyperspectral	Potato multi-stage experiment	AGB R^2^ = 0.81	Pre-harvest yield forecasting	[189]
Soil quality assessment	Field observations + multi-temporal RS + LULC	Hainan Island; 152 sites; 2021–2024 RS data	SRSI r = 0.781; SI r = 0.663	Precision agriculture and soil management	[190]
Agricultural machinery localization	LiDAR + GNSS + IMU	Machinery sheds and farm access roads	APE reduction: 79.99% and 49.04%; error: 3.97 cm	Autonomous navigation and control	[191]
Machinery optimization	WOA + LIBSVM classifier	Harvester vibration tests	Fault diagnosis accuracy > 90%	Vibrating screen repair schedule	[192]
	PSO + wavelet neural network	Combine harvester experiments	VAF ≥ 81.7%; RMSE ≤ 0.602	Forward speed and fan airflow tuning	[193]

## Data Availability

Data availability is not applicable to this article as no new data were created or analyzed in this study.
